# Biogenesis, Stabilization, and Transport of microRNAs in Kidney Health and Disease

**DOI:** 10.3390/ncrna4040030

**Published:** 2018-11-03

**Authors:** Melissa J. Thomas, Donald J. Fraser, Timothy Bowen

**Affiliations:** Wales Kidney Research Unit, Division of Infection and Immunity, School of Medicine, College of Biomedical and Life Sciences, Cardiff University, Cardiff CF14 4XN, UK; Thomasmj11@cf.ac.uk (M.J.T.); fraserdj@cf.ac.uk (D.J.F.)

**Keywords:** microRNA, extracellular vesicle, chronic kidney disease

## Abstract

The kidneys play key roles in the maintenance of homeostasis, including fluid balance, blood filtration, erythropoiesis and hormone production. Disease-driven perturbation of renal function therefore has profound pathological effects, and chronic kidney disease is a leading cause of morbidity and mortality worldwide. Successive annual increases in global chronic kidney disease patient numbers in part reflect upward trends for predisposing factors, including diabetes, obesity, hypertension, cardiovascular disease and population age. Each kidney typically possesses more than one million functional units called nephrons, and each nephron is divided into several discrete domains with distinct cellular and functional characteristics. A number of recent analyses have suggested that signaling between these nephron regions may be mediated by microRNAs. For this to be the case, several conditions must be fulfilled: (i) microRNAs must be released by upstream cells into the ultrafiltrate; (ii) these microRNAs must be packaged protectively to reach downstream cells intact; (iii) these packaged microRNAs must be taken up by downstream recipient cells without functional inhibition. This review will examine the evidence for each of these hypotheses and discuss the possibility that this signaling process might mediate pathological effects.

## 1. Introduction

### The Kidneys

The kidneys are a pair of bean-shaped organs located within the retroperitoneal space, either side of the spinal column. The left kidney is situated between vertebral levels T12 to L3, with the right kidney resting slightly inferior, due to displacement by the liver [1]. In human adult males, each kidney is approximately 11 cm in length, and weighs approximately 150 g.

Renal functions in the maintenance of homeostasis include the regulation of acid-base balance, osmolality, blood pressure and extracellular fluid volume. In addition, the kidneys produce hormones, including calcitriol, angiotensin, and aldosterone.

**Figure 1:**
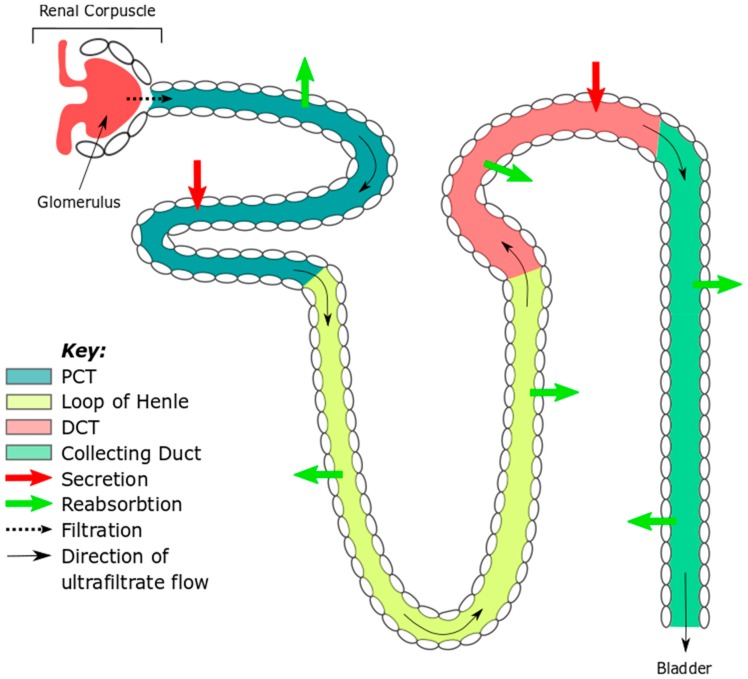
The nephron—the functional unit of the kidney. A different color is used to highlight each nephron domain. The direction of ultrafiltrate flow is shown with black arrows, bold arrows signify secretion of waste products (red) and solute reabsorption (green). PCT, proximal convoluted tubule; DCT, distal convoluted tubule.

The functional unit of the kidney is the nephron (Figure 1). Each kidney possesses approximately 1.3 million nephrons and each nephron is composed of several regions: The Bowman’s capsule that is intimately associated with the glomerulus in the renal corpuscle; the proximal convoluted tubule (PCT); the loop of Henlé; the distal convoluted tubule (DCT) and the collecting duct.

Blood filtration takes place in the Bowman’s capsule. The PCT, loop of Henlé, DCT and collecting duct are concerned with selective solute reabsorption and secretion of waste components between the ultrafiltrate and the circulation. The ultrafiltrate then leaves the collecting duct en route to the renal papilla and then the bladder, from which it is excreted as urine. This direction of flow from glomerulus through the nephron dictates the course taken by ultrafiltrate-borne microRNAs (miRNAs) and of any signaling mediated by these transcripts.

Underlining the importance of the kidneys’ physiological roles during homeostasis, renal pathologies, such as chronic kidney disease, diabetic kidney disease, acute kidney injury, renal cancer, glomerulonephritis, and polycystic kidney disease, lead to widespread morbidity and mortality that place a significant burden on health services worldwide.

A more complete understanding of the processes underlying communication within the kidney promises to provide novel targets for disease prevention and treatment strategies [2]. Here we will review the evidence for miRNA-mediated intra-nephron signaling and for pathological effects mediated by this mechanism.

## 2. microRNAs

### 2.1. Discovery and Evolutionary Conservation of microRNAs

microRNAs were first identified in 1993 [3,4] and are found in algae, viruses, plants, invertebrates, and vertebrates [5,6,7]. miRNA-mediated silencing mechanisms show ancient evolutionary origins [8]. The sequences that give rise to miRNAs may be located in introns of protein coding genes, in exons and introns of long noncoding RNAs and in intergenic regions [9]. miRNAs are single-stranded RNA transcripts most often of ~22 nucleotides in length, with strong secondary structure motifs. Mature miRNAs may be clustered into families based on their nucleotide sequences [5].

### 2.2. Biogenesis of miRNAs

**Figure 2:**
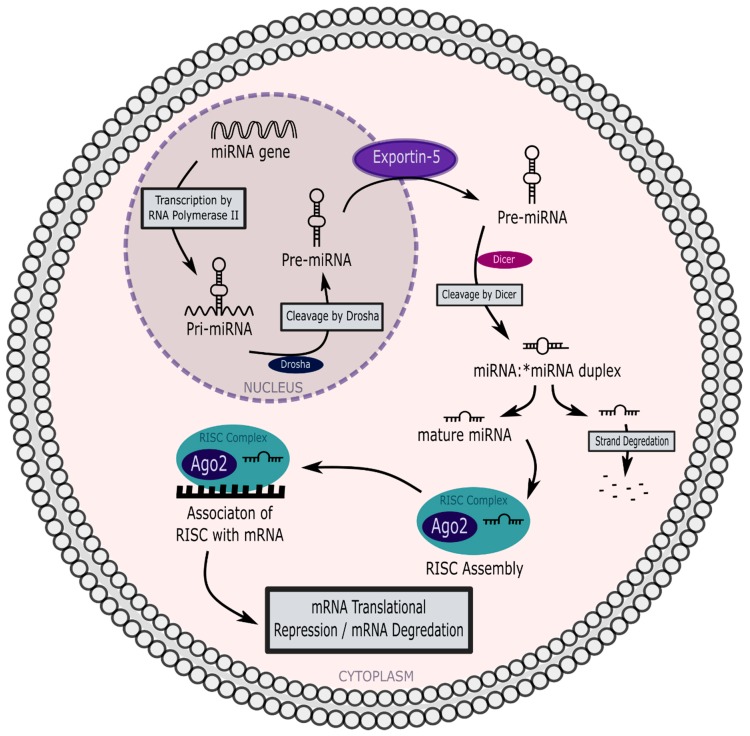
Nuclear transcription and export of microRNAs (miRNAs), and their roles in translational repression. RISC, RNA-induced silencing complex.

As summarized in Figure 2 below, most miRNAs are transcribed by RNA polymerase II as primary miRNAs (pri-miRNAs) that exceed 200 nucleotides in length. Pri-miRNAs are next cleaved into 60–70 nucleotide hairpin precursor miRNAs (pre-miRNAs) by the Microprocessor multiprotein complex, a dimer composed of RNAse III enzyme Drosha and double-stranded RNA binding protein Pasha/DGCR8.

Pre-miRNAs are exported from the nucleus to the cytoplasm by Exportin-5, where they are processed by a second RNAse III enzyme, Dicer. Dicer cleavage results in the formation of mature ~22 nucleotide long miRNA/miRNA* duplexes with a guide strand and a passenger strand, denoted here by an asterisk, which is degraded when the duplex divides. The guide strand associates with several RNA binding proteins, including argonaute 2 (Ago2), to form the microribonuclear protein (miRNP) complex known as the RNA-induced silencing complex (RISC) (Figure 2). The mature miRNA may silence target gene expression via two mechanisms: Binding to a target messenger RNA (mRNA) strand thereby preventing its translation and/or through promoting target mRNA degradation. Biogenesis of miRNAs is reviewed in detail elsewhere [10].

By widespread regulation of target mRNA translation and subsequent protein expression, miRNAs help modulate the physiological processes that maintain homeostasis. Dysregulated miRNA expression has been reported in the pathophysiology of numerous diseases, including malignancies and cardiovascular disease [11].

### 2.3. microRNAs in Kidney Health and Disease

Key roles for miRNAs have been reported in all types of renal cell, demonstrating their importance in kidney development and the maintenance of homeostatic kidney physiology [12]. Aberrant miRNA expression has been observed in renal diseases, including kidney cancer, acute kidney injury, end-stage renal disease, diabetic nephropathy and polycystic kidney disease [13,14,15,16,17,18,19,20].

As described above, Dicer processes pre-miRNAs to mature miRNAs in canonical miRNA biogenesis, but non-canonical miRNA synthesis pathways have also been described [21,22]. Germline Dicer knockout in mice results in non-viability, reflecting the crucial importance of miRNAs in development [23]. In the kidney, podocyte-specific Dicer knockout results in glomerular and proximal tubular injury with accompanying proteinuria [24,25,26] with similar results for Drosha ablation [27]. By contrast, it has been reported that proximal tubular-specific Dicer knockouts result in normal renal function and protection against renal ischemia-related injury, although in this model significant residual miRNA expression was quantifiable [28]. Dicer’s miRNA-independent roles [29,30] might also complicate interpretation of data from models employing Dicer deletion.

In order to propose miRNAs as communicators between segments of the nephron, renal cells must demonstrate the capacity to release miRNAs, package them appropriately for protected transport through the ultrafiltrate, and to take up functional miRNAs from the extracellular environment, which are able to exert a phenotypic effect on the recipient cell.

Table 1 below presents data on selected miRNAs in kidney health and disease. We apologize to those authors whose work we were unable to include, due to the volume of publications in this area. Excellent and comprehensive recent reviews covering this area include Rong et al. [31], and Gomez et al. [32].

## 3. Intra-Nephron microRNA Transport

### 3.1. Extracellular Vesicle Nomenclature

As discussed below, many reports describe the presence of extracellular vesicle-associated and non-extracellular vesicle-associated miRNAs in body fluids. For the purposes of this review, wherever possible we will use the definitions from the recent comprehensive review by van Niel and colleagues [48]. Briefly, when extracellular vesicles (EVs) are formed by plasma membrane budding they are referred to as microvesicles (MVs), and fall typically within the 50–500 nm size range, but may be as large as 1 µm. A second population of EVs is generated within the lumen of multivesicular endosomes. Fusion of these endosomes with the plasma membrane results in the release of this second EV population, which are referred to as exosomes and typically range in size from 50–150 nm [48]. Nevertheless, the reader should be aware of significant inconsistencies in past use of the terms EV, microvesicle and exosome, and the wide variety of methods that have been used in EV isolation. Further details are not within the remit of this review and may be obtained from the cited sources.

**Hypothesis** **1.** *miRNAs are released into the upstream ultrafiltrate*.

### 3.2. Cellular Release of microRNAs

To date, comparatively little data on intra-renal miRNA transport have been reported. However, numerous cell types release EVs, including dendritic cells [49], lymphocytes [50,51], endothelial cells [52,53], mast cells [54], epithelial cells [55] and tumor cells [56]. Consequently, EVs have been found in a range of body fluids, including saliva [57], blood plasma [58], cerebrospinal fluid [59], amniotic fluid [60], pleural fluid [61], and urine [62]. The work of Valadi and colleagues first described the presence of RNAs, including miRNAs, in exosomes released by mast cells [63].

Proteomic profiling of human urinary exosomes has revealed the presence of proteins specifically expressed in the following nephron segments: Glomerular podocytes, proximal tubule, thick ascending limb of Henle, distal convoluted tubule, collecting duct, and transitional epithelia from the urinary drainage system [60,62,64,65].

The above data support the hypothesis that renal cells release exosomes into the ultrafiltrate.

**Hypothesis** **2.** *Released miRNAs are sufficiently stable to reach downstream cells intact*.

### 3.3. Extracellular microRNA Stability

**Figure 3:**
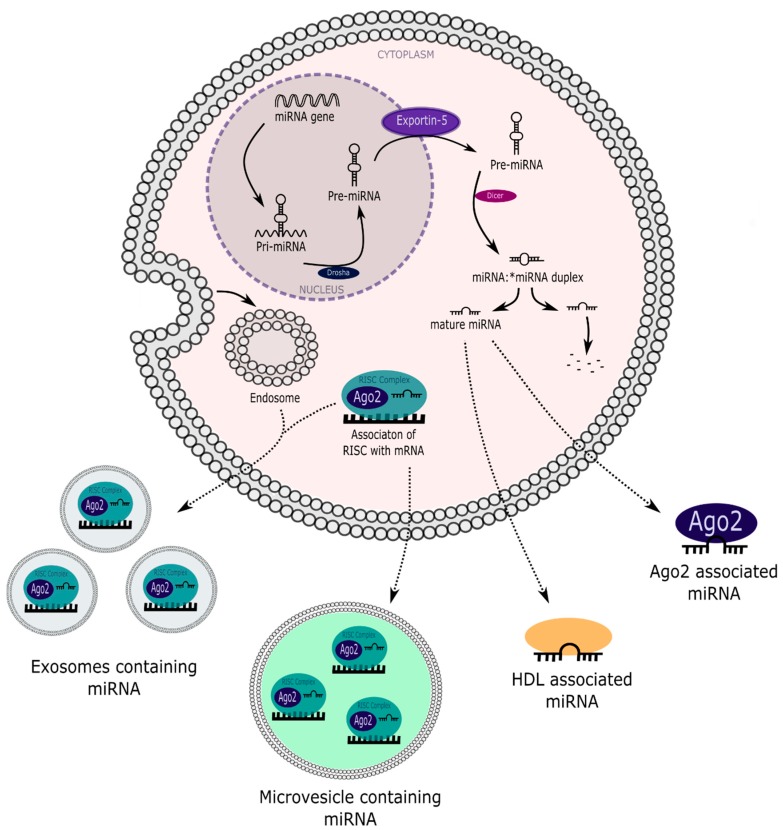
miRNA release mechanisms. HDLs, high-density lipoproteins; pre-miRNAs, precursor miRNAs; pri-miRNAs, primary miRNAs.

As part of immune surveillance against viral infection, biological fluids, including urine, contain highly active RNA-degrading ribonucleases [66,67,68]. Consistent with this, we found that synthetic *Caenorhabditis elegans* miR-39 added to human urine was degraded very rapidly [69]. By contrast, stability of cell-free endogenous miRNAs has been demonstrated in plasma, serum, urine and tissue culture medium, suggesting protection from endogenous ribonucleases [69,70,71,72,73,74]. miRNAs in the extracellular space may be stabilized by association with EVs [69,75] (Figure 3), and a recent study has comprehensively demonstrated the stability of EV-associated circulating miRNAs [76].

However, extracellular miRNAs do not associate exclusively with EVs (Figure 3). Wang and co-workers [77] exposed human cells to acute stress and analysed the culture medium. Subsequent differential centrifugation revealed the presence of miRNAs in centrifugation pellets containing EVs (referred to hereafter as EV-associated or EVA), and EV-free fractions (non-EVA). Supporting these findings, a study by Arroyo et al. [72] reported two distinct populations of plasma-borne miRNAs: EVA-miRNAs associated with vesicular ultracentrifugation fractions collected with painstaking precision to avoid EV rupture, and non-EVA-miRNAs.

This latter study also reported that the majority of plasma miRNAs were non-EVA-miRNAs associated with RISC component Ago2 (Figure 2) [72]. Association of plasma non-EVA-miRNAs with Ago1 [78] and Ago2 was also reported elsewhere [73,78]. Western blot analysis of both plasma and conditioned cell culture media following ultrafiltration showed association of most non-EVA-miRNAs with Ago2 [73], which is believed to confer stability and protection from degradation [69,72,79].

Our laboratory is investigating the use of miRNAs in urine and other body fluids as kidney disease biomarkers [14,18,19,20,34,80,81,82]. On the basis of the above studies, we analyzed human urine for presence on EVA and non-EVA-miRNAs [69]. Using established and optimized ultracentrifugation protocols for isolation of intact EVs [83], we showed association of miR-16 and miR-192 with exosomal and non-exosomal EV fractions [69]. We then used RNA-immunoprecipitation to show association of these miRNAs with AGO2 [69].

High-density lipoproteins (HDLs) have also been implicated in the transport of miRNAs in the extracellular circulatory environment [84]. This relationship was first proposed following the finding that purified HDL fractions from human plasma contained miRNAs [84]. Transmission electron microscopy allowed visualization of immunoprecipitated miRNA-HDL complexes that were clearly distinguishable from EVs [84]. This association has the potential to protect miRNAs from ribonuclease activity, and these authors proposed that miRNA-HDL transport represented an alternative form of intercellular signaling [84] a theme that has attracted considerable further attention [85]. Low-density lipoprotein (LDL) fractions from human plasma also contain miRNAs, but LDLs are less robust miRNA carriers than HDLs [84,85]. Consequently, LDLs have received less attention in the context of miRNA transport. A new pipeline for systematic analysis of lipoprotein-associated miRNAs has been developed to expedite acquisition of this knowledge [86]. HDLs and LDLs are too large to pass through the glomerular filtration barrier into the ultrafiltrate, and so are not predicted to play a part in intraluminal miRNA transport within the nephron. However, it is conceivable that other, as yet undiscovered, miRNA chaperones may be found in the ultrafiltrate.

Collectively, the above corroborate the hypothesis that miRNAs leaving nephron cells are protected sufficiently from endogenous urinary ribonucleases.

**Hypothesis** **3.** *Downstream cells take up functional miRNAs from the ultrafiltrate*.

### 3.4. Downstream Uptake of microRNAs

To date, much of the analysis of miRNA cellular uptake has focused on EVA-miRNAs. The process of EV binding to target cells is likely directed by recipient cell surface receptors and EV membrane protein composition: Following binding, internalization by endocytosis may be clathrin-mediated or -independent, vesicular fate is dictated by their composition and target cell plasma membrane structure. Once EVs have fused with the recipient cell, they elicit functional responses by receptor activation at the recipient cell surface, and EV-miRNA and mRNA cargoes can activate responses following internalization [48,63,87]. While the process of miRNA extracellular transport is now widely accepted, the active/passive components of EV miRNA loading remain unresolved [88].

Exosomes were first implicated in the mediation of cell-to-cell communication via antigen presentation [51]. Valadi and colleagues [63] subsequently showed the presence of exosome-associated mRNAs and miRNAs from human and mast cell lines, and primary mouse mast cells. These authors demonstrated de novo protein synthesis from transferred mRNA, suggesting that this represented a novel mechanism of inter-cellular genetic exchange [63]. The concept of exosomes as novel mediators of horizontal genetic transfer between cells soon expanded to include miRNAs synthesized in response to viral infection, and mitochondrial DNA [89,90].

Numerous studies have described the regulation of target genes by EV-transported miRNAs. Delivery of miR-126 in endothelial EVs derived from human aortic smooth muscle cells (HASMCs) targeted regulator of G protein signaling 16 (RGS16) following transfer to human umbilical vein endothelial cells (HUVECs), thereby inducing CXCL12 expression via CXCR4 [91]. Collino and colleagues [92] showed that MVs delivered endogenous and synthetic miRNAs, and noted downregulated expression of phosphatase and tensin homologue (PTEN), cyclin D1 and B cell lymphoma 2 (Bcl-2), which they attributed to transferred miRNAs [92].

Zhang and co-workers [93] transfected fluorescently tagged synthetic miR-150 into THP-1 cells. MVs subsequently isolated from these cellswere then added to human dermal microvascular endothelial cells and miR-150 transfer was observed, which resulted in reduced protein levels of oncoprotein c-Myb. Communication between endothelial and HASMCs has been reported to confer an atheroprotective effect [94]. Transcription factor Krüppel-like factor 2 (KLF2) induces expression of miR-143 and miR-145. KLF2-transduced HUVECs produced EVs enriched in these miRNAs, and subsequent co-culture with untreated HASMCs resulted in significantly decreased HASMC expression of 6 miRNA target genes, including ETS transcription factor family protein ELK1 and matrix metalloproteinase 3 (MMP3) [94]. A further study has shown reduced adipogenesis and lipogenesis in porcine adipocytes as a result of PPAR-γ repression, driven by EV-shuttled miR-130b [95].

Of direct relevance to the kidney, a recent study presents time-lapse video evidence showing EVs moving into renal proximal tubule cells by EV uptake, and also reports EV uptake by renal distal tubule cells and collecting duct cells [96]. These authors also present evidence for functional transfer of PTC EVs to distal tubule and collecting duct cells, positing that this provided proof of a proximal-to-distal intra-nephron transfer between upstream proximal tubular cells and downstream recipients [96]. Furthermore, recent sequence analysis has identified 276 mature miRNAs in urinary exosomes from healthy subjects and observed enrichment of miR-10, miR-30 and let-7 families [97]. Cultured cells from human renal proximal tubular cell line HKC-8 were then seen to take up urinary exosomes, which was followed by translational repression of potassium channel ROMK and kinases SG1 and WNK1 [97].

Taken together, these studies provide strong supportive evidence for the uptake and function of EVA-miRNAs by renal and other cells. Comparatively little attention has so far been paid to transfer of functional non-EVA-miRNAs. As discussed above, there is strong evidence for HDL-mediated miRNA transport in the circulation, and non-EVA-miRNAs are readily detectable in urine. The potential for alternate non-EVA-miRNA transport and signaling mechanisms in paracrine miRNA signaling within the nephron is an important area for future study.

## 4. Conclusions

The experimental studies detailed above provide strong support for the hypothesis that endogenous miRNAs function as intra-nephron communicators. Key roles for miRNAs in disease processes in the kidney are emerging. A complete understanding of intra-nephron miRNA transport and function might permit the use of relevant miRNAs as biomarkers. Such biomarkers are intended to provide a non-invasive method of measuring response to treatment in a patient with kidney disease, or a mechanism by which likely response to treatment could be predicted. MiRNAs are also entering testing as direct targets of therapy, and as a potential therapy approach themselves. Understanding the mechanisms by which miRNAs are protected, packaged and taken up by target cells in the nephron may provide valuable insights for novel approaches to miRNA therapy.

## 5. Patents

T.B. and D.J.F. are inventors for patent WO/2017/129977 Chronic Kidney Disease Diagnostic.

## Figures and Tables

**Table 1:** MiRNAs implicated in kidney disease pathologies. MCs, mesangial cells. DN, diabetic nephropathy.

microRNA	Up/Down-Regulation in Disease/Model	Identified Target	Disease/Model	References
MiR-192	Up	SIP-1	Mouse Model, MCs (human, rat, mouse)	[33]
	Down	Zeb2	DN patient samples, Proximal Tubule cells	[34,35]
MiR-29c	Up	Sprouty homolog-1/HIF1α	Mouse Model, MCs (mouse)	[36,37]
MiR-21	Up	Smad7	Mouse Model, MCs (rat)	[38]
	Up	PPARa	Human Kidney Biopsy	[39]
	Up	MMP-9, TIMP1	Mouse Model, MCs (rat)	[40]
	Down	PTEN	Mouse Model, Primary MCs (mouse)	[41]
MiR-215	Up	CTNNBIP1	Mouse Model, Primary MCs (mouse)	[37]
	Down	Zeb2	Mouse Model, Primary MCs and PTCs (rat)	[35]
MiR-200b/c	Up	Zeb1/2	Mouse Model, MCs (mouse), Endothelial cells	[42,43]
MiR-29b	Down	TGFBR	Mouse Model	[44]
MiR-216a	Up	PTEN Ybx1	Mouse Model, Primary MCs (mouse)	[45]
MiR-25	Down	NOX4	Human Biopsy, MCs	[46]
MiR-29a	Down	COL4α1/2	Proximal Tubule cells	[47]
